# Dual-Beam Photothermal Spectroscopy Employing a Mach–Zehnder Interferometer and an External Cavity Quantum Cascade Laser for Detection of Water Traces in Organic Solvents

**DOI:** 10.1021/acs.analchem.2c03303

**Published:** 2022-11-16

**Authors:** Giovanna Ricchiuti, Alicja Dabrowska, Davide Pinto, Georg Ramer, Bernhard Lendl

**Affiliations:** Institute of Chemical Technologies and Analytics, TU Wien, Getreidemarkt 9/164-UPA, Vienna1060, Austria

## Abstract

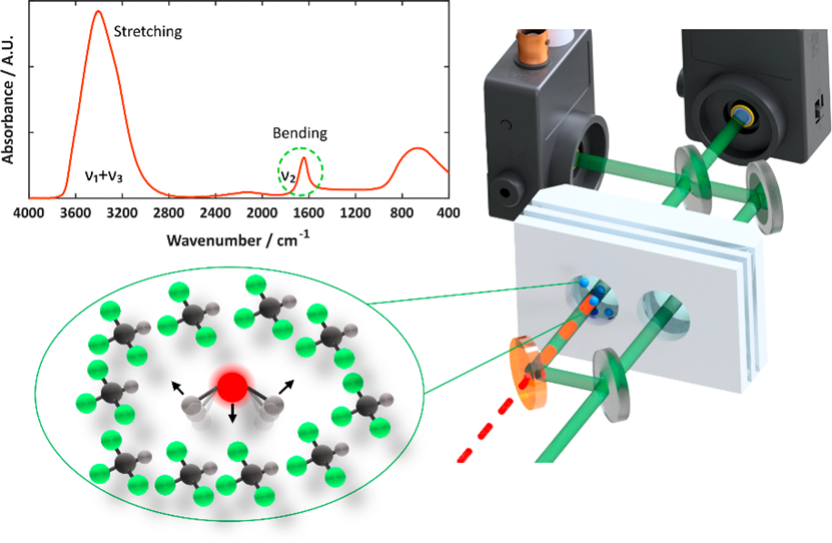

We report on a mid-infrared (mid-IR) photothermal spectrometer for liquid-phase samples for the detection of water in organic solvents, such as ethanol or chloroform, and in complex mixtures, such as jet fuel. The spectrometer is based on a Mach–Zehnder interferometer (MZI) employing a He-Ne laser, a mini-flow cell with two embedded channels placed in the interferometer’s arms, and a tunable external cavity quantum cascade laser (EC-QCL) for selective analyte excitation in a collinear arrangement. In this study, the bending vibration of water in the spectral range 1565–1725 cm^–1^ is targeted. The interferometer is locked to its quadrature point (QP) for most stable and automated operation. It provides a linear response with respect to the water content in the studied solvents and photothermal analyte spectra, which are in good agreement with FTIR absorbance spectra. The method is calibrated and validated against coulometric Karl Fischer (KF) titration, showing comparable performance and sensitivity. Limits of detection (LODs) for water detection in the single-digit ppm range were obtained for chloroform and jet fuel due to their low background absorption, whereas lower sensitivity has been observed for water detection in ethanol due to pronounced background absorption from the solvent. In contrast to KF titration, which requires toxic reagents and produces waste, the developed method works reagent-free. It can be applied in an online format in the chemical industry as well as for fuel quality control, being industrial applications where traces of water need to be accurately determined, preferably in real-time. It thus holds great promise as a green alternative to the offline KF titration method, which is the current standard method for this application.

Mid-infrared (mid-IR) spectroscopy (400–4000 cm^–1^) stands out as a versatile analytical technique that allows for qualitative and quantitative analysis as well as chemical imaging. It is based on probing highly selective rotational and vibrational transitions of analyte molecules present in gases, liquids, and solids. Information on the energies of these transitions is given by the wavelength of the absorbed mid-IR radiation, providing direct access to molecular specific information.^1^ Analytical methods based on mid-IR spectroscopy are often conceptionally simple, as in many cases, only little or even no sample preparation is required. This has led to a widespread use of this technique in many different application areas. The most common types of mid-IR spectrometers in use today are equipped with a highly stable but rather weak thermal light source (globar) and make use of an interferometer to guarantee high energy throughput. Maximum spectral power densities found in the sample compartment of FTIR spectrometers are typically below 20 μW/cm^–1^.^2^ For demanding applications and when seeking highest sensitivities, these spectrometers are usually equipped with a liquid-nitrogen-cooled mercury cadmium telluride (MCT) detector.^3^

For the measurement of liquids, absorbance measurements are carried out using either transmission cells with pathlength in the micrometer range or different variants of evanescent wave sensing mainly employing the so-called attenuated total reflection (ATR) technique.^4^ In either case, background and sample single beam spectra are needed for calculation of the absorbance spectrum of the sample under investigation.^3^ Whereas there is a linear relation between the absorbance and concentration, it has to be stressed that the recorded intensities decrease exponentially with increasing analyte concentration and pathlength as per the Beer–Lambert law:^5^
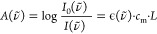
1

In recent years, room temperature-operated, small but powerful mid-IR lasers operated in pulsed as well as CW mode have become commercially available from different providers, and as such they have been used in mid-IR sensing applications.^6^ QCLs equipped with an external diffraction grating, called external cavity (EC), allow for a broad tunability across the mid-IR range (a few hundred wavenumbers) that can be particularly exploited for liquid-phase analytes.^6−9^ The favorable properties of these sources are high spectral power densities up to several hundreds of milliwatts in terms of peak power as well as several tens of milliwatts in CW operation. Furthermore, coherence, inherent polarization, and the possibility for fast albeit accurate amplitude and frequency modulation offer opportunities to develop new sensing schemes.

It is interesting to note that many of the recent breakthrough developments in mid-IR-based sensing, especially in the fields of photoacoustic^10^ or photothermal^11^ gas sensing as well as IR imaging beyond the diffraction limit,^12^ make use of indirect measurement concepts. In these, the photon energy, which has been initially absorbed by the analyte molecule is released into the sample matrix, causing a slight increase in its temperature. Compared to established absorbance spectroscopy, several remarkable differences can be identified. The magnitude of the temperature-induced signal is directly proportional to laser power *P*(ν̃), the analyte linear absorption coefficient α(ν̃), the optical pathlength *L* and indirectly with respect to the product of the density ρ and heat capacity *C*_P_ of the sample, the applied modulation frequency *f*_mod_, as well as the volume of the beam when interacting with the sample *V*.^13−15^
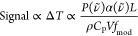
2

As a result, the sensitivity of indirect techniques increases directly proportional with the laser power *P*(ν̃) and also upon miniaturization by reducing the volume of interaction *V*, a stark difference to absorbance spectroscopy based on the Beer–Lambert law, where the pathlength *L* has to be enlarged for increased sensitivity.

In a feasibility study,^16^ we have previously demonstrated a first photothermal mid-IR setup based on a Mach–Zehnder interferometer (MZI) for the analysis of liquids by reporting on successful detection of caffeine in ethanol and highlighting encountered difficulties when aiming to detect proteins in aqueous solutions. Now, we are able to report on an advanced MZI setup with significantly improved stability due to an implemented locking scheme and on the successful application of the developed system to a real-world analytical problem being the sensitive detection of water traces in organic solvents at low ppm concentration levels.

The developed setup allows for direct, rapid, and nondestructive measurements. It provides an alternative to the Karl Fischer (KF) titration method, which is the gold standard for determining water traces in organic solvents but is characterized by the usage of expensive and toxic reagents that lead to sample consumption and waste generation. The KF titration method is generally used offline and thus not suited for real-time monitoring^17^ of industrial processes in the chemical industry where water traces represent a major concern especially when absolute solvents are required in chemical synthesis.^18^ A further need for detecting trace water levels exists in the aviation industry. Water from different sources may easily contaminate jet fuel because of the latter’s hygroscopic nature. For safety reasons, the water content in jet fuel must be kept within certain limits to ensure compliance with standards allowing a maximum of 90 ppm-v for normal system operation and 260 ppm-v for emergency system operation.^19^ Therefore, there is a need for chemical sensors that are able to quantify minute amounts of water in organic solvents and jet fuel which have the prospect of being implemented in an online or even inline configuration.^20^ A comparison of alternatives to the KF titration method for trace water analysis in terms of reliability, accuracy, limit of detection (LOD), and stating advantages as well as drawbacks was recently compiled by Kumar et al.^21^

The mid-IR spectrum of water is characterized by its unique fingerprint comprising three fundamental molecular vibrations: the antisymmetric O–H stretching vibration (ν_3_ ∼ 3500 cm^–1^), the symmetric O–H stretching vibration (ν_1_ ∼ 3300 cm^–1^), and the H–O–H bending vibration (ν_2_ ∼ 1645 cm^–1^). In addition, several libration modes around 600 to 800 cm^–1^ and a combination band at 2130 cm^–1^ can be observed in the mid-IR spectrum of liquid water.^22^ The infrared spectrum of pure water as well as of mixtures with other solvents has been investigated in detail already. Most work has focused on changes in band shape and band position of the O–H stretching modes, which are strongly affected by intermolecular interactions, such as H-bonding. Only recently, also the bending mode of water (ν_2_ ∼ 1645 cm^–1^), which is under investigation in this work, has been investigated in detail and identified as a powerful probe for hydrogen bond structure of aqueous systems.^23,24^ Because our setup uses a tunable mid-IR laser for sample interrogation, it is also sensitive to the chemical environment of water molecules in the sample.

In this study, we describe the developed setup and give examples on trace water detection in a protic and an aprotic solvent, as well as in jet fuel. The optimized setup has the capability to determine water concentrations in the low ppm concentration range as corroborated by reference analysis carried out by coulometric KF titrations. Finally, we show that the obtained spectra of water in the investigated solvents and in jet fuel agree well with reference spectra recorded on an FTIR spectrometer, highlighting the key role and influence of the solvent in the analyte detection.

## Experimental Section

### Reagents and Samples

Ethanol absolute, anhydrous (max. 0.003% H_2_O) ≥99.8%, was purchased from VWR Chemicals (Vienna, Austria). Proper amounts of ultrapure water (resistivity: 18 MΩ cm) were dissolved in ethanol to create solutions of different concentrations. Chloroform, anhydrous (max. 0.005% H_2_O) stabilized, was purchased from VWR Chemicals. Type A-1 jet fuel as used in aviation industry was provided by a local refinery (OMV Refining & Marketing GmbH, Vienna, Austria).

Aliquots (∼100 mL) of chloroform and jet fuel were mixed with molecular sieve pearls (molsieve) to yield dried samples. Furthermore, aliquots (∼100 mL) of chloroform and jet fuel were mixed with ultrapure water, sonicated, and left overnight in a separatory funnel for phase separation yielding samples with maximum water content. From the so-prepared samples, (chloroform/jet fuel + water) and (chloroform/jet fuel + molsieve), two standards of different water content in jet fuel and chloroform were prepared by mixing. The prepared standards were first used for establishing the calibration curves and analyzed immediately afterward for their water content using the KF titrator to avoid environmental/storage contamination.

It is worth highlighting that the study does not aim at performing measurements under ultra-dry conditions, which would require special measures (glove box and vacuum) to prevent sample contamination during sample preparation and measurement. The main applied goal of this study is to demonstrate successful determination of the water content of organic solvents and jet fuel under typical laboratory conditions as encountered in routine operations.

### Spectrometer for Photothermal Spectroscopy Based on an MZI (PTS-MZI Spectrometer)

**Figure 1:**
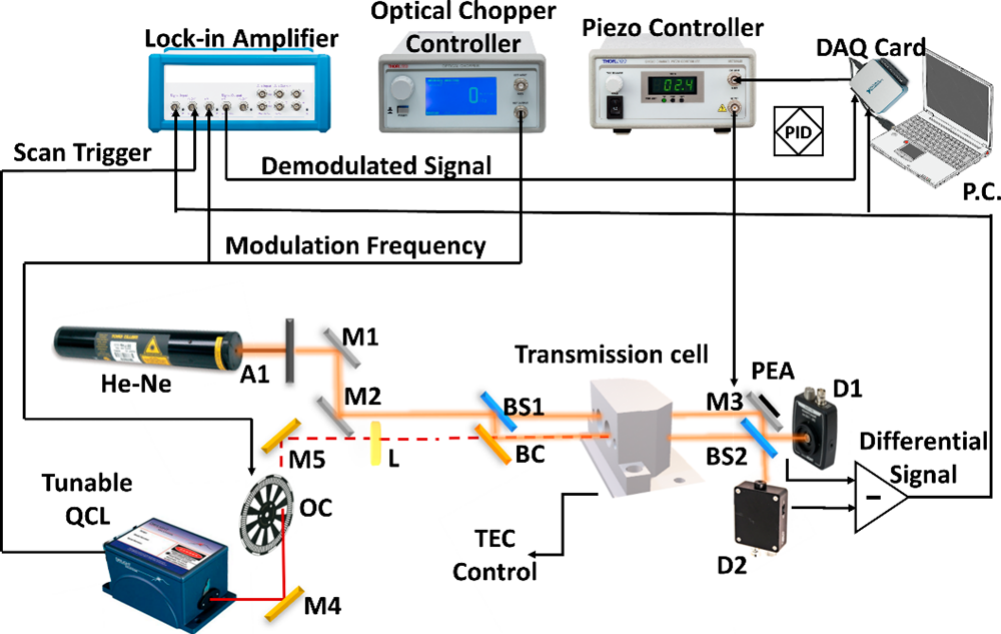
Schematic of pump-probe photothermal Mach–Zehnder interferometer setup for liquid-phase samples spectroscopy. (M1–5...mirrors, L...lens, A1...attenuator, OC...optical chopper, BS1–2...beam splitter, BC...beam combiner, D1–2...Si detectors, and PEA...piezo electric actuator).

The basic principle of the PTS-MZI spectrometer has already been described in our previous study.^16^Figure 1 reports the schematic of the setup used in this study. A commercially available external cavity quantum cascade laser (EC-QCL) (DRS Daylight Solutions Inc., San Diego, CA, USA) is used as a pump source. It is operated in CW mode with a laser current of 650 mA. The operating temperature of the water-cooled laser head is set to 20 °C. The excitation source is modulated by means of an optical chopper with a duty cycle of 50% (Thorlabs, MC2000B-EC, Blade: MC1F10HP).

The probe source is a linearly polarized helium–neon laser (Melles Griot, Rochester, NY, USA) with an emission wavelength of 632.8 nm. An attenuator (Thorlabs, PVAE1-A) is introduced immediately at the output of the visible beam to reduce the brightness coming from the source and to avoid saturation of the two photodetectors (DET10A, Thorlabs, Bergkirchen, Germany), allowing for linear response. The attenuated red light is split at a ratio of 50:50 (R:T) by means of a UV-fused silica beam splitter (Thorlabs, BSW04). One beam is redirected through the reference arm of the MZI, while the second beam is combined coaxially with the mid-IR excitation beam, in the analyte channel of the interferometer, via a custom-made ZnSe beam combiner with a maximum reflection *R*_max_ at 633 nm and maximum transmission *T*_max_ at 10.6 μm (Laser Components, Olching, Germany). The visible beams are then recombined via a second, identical beam splitter. Two additional attenuators (Thorlabs, PVAE1-A) are included immediately before the two Si detectors to adjust for uniform maximum response. One of the mirrors in the interferometer is mounted to a chip piezo electric actuator (Thorlabs, PA4FKW) connected to a commercial piezo controller (Thorlabs, MDT694B), allowing precise automated adjustment of the interferometer beam paths. The optical components of the experimental setup are placed on a water-cooled breadboard (Thorlabs, MBC3045/M) combined with a liquid-cooling system (ThermoCube, Solid State Cooling Systems, Wappingers Fall, NY, USA) for additional stabilization through temperature control (22.5 °C). Moreover, a dedicated black-walled housing is used as enclosure for the spectrometer, and the system is continuously flushed with dry air to reduce water vapor contribution, as a source of noise, in the spectral region under investigation.

### Sample Handling and Injection System

The liquid samples are injected in a temperature-stabilized (22.5 °C) transmission mini-flow cell. It consists of a block that includes two channels (one for the reference and one for the analyte), placed in the middle of the interferometer, with a PTFE spacer (110 μm) defining the pathlength between two wedged CaF_2_ windows. Each channel of the transmission cell has one inlet and one outlet. To remove the unknown amount of water in the sample, an in situ sample injection and drying system has been developed. In doing so, it is possible to prepare a dried reference sample in situ.

**Figure 2:**
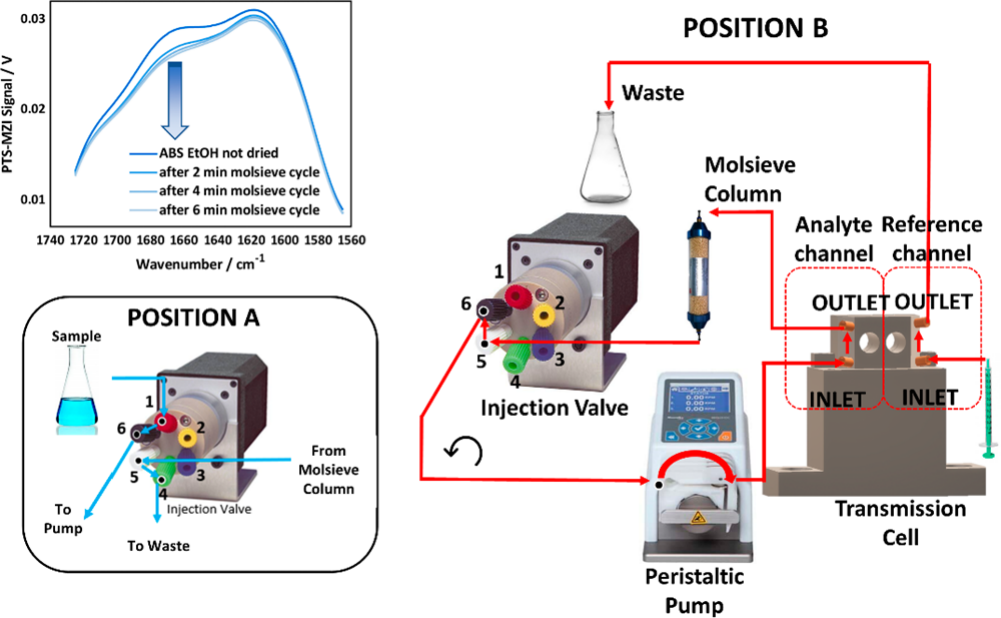
Schematic of in situ reference to dry the solvents during the measurement. Position A permits the injection of the sample through the analyte channel. Position B permits to close the loop to circulate the injected sample through the molecular sieve column, drying it.

The system, reported in Figure 2, consists of a 6-port injection valve (Cheminert 6-port, VICI, Schenkon, Switzerland) connected to a peristaltic pump (Ismatec Reglo, Ismatec, Wertheim, Germany). The output of the peristaltic pump is connected to the inlet of the analyte channel of the transmission cell. A column (radius: 0.62 mm, length: 7 cm) filled with 6–7 g molecular sieve, previously dried at 350 °C for 24 h, is placed at the output of the analyte channel. A dried liquid sample is also injected in the reference channel via a syringe. The valve allows to switch between two configurations: position A and position B.

Prior to measurement, the valve is set to position A (left-lower inset in Figure 2). The solvent flows from port 1 to port 6, passing the pump to the cell and through the molecular sieve column. The solvent then reaches port 5, and moving across port 4, it is directed to the waste. By this, the analyte channel is filled and the valve is switched to position B to close the loop. The solvent can now be circulated through the column for the time needed to remove the unknown water content. A 3 Å molecular sieve is used to dry both ethanol and jet fuel, while a 4 Å molecular sieve is employed to dry chloroform.

Once the spectra recorded during the drying process no longer change, the solvent is dried to the best of the system’s capability. For example, as per the left-upper inset graph in Figure 2, in the case of ethanol, 5 to 6 min are necessary to dry the injected volume. The last acquired spectrum (*I*_back_) is used for background correction as per eq 3.

### Signal Acquisition

As per Figure 1, the differential signal (D1-D2) coming from the two photodetectors (D1 and D2) is sent to the lock-in amplifier (LIA) (MFLI, Zurich Instruments, Zurich, Switzerland) as an input. The LIA demodulates the differential signal using the chosen optical chopper modulation frequency *f*_mod_ as a reference. Data acquisition uses the “Forward Sweep Scan” mode clock of the EC-QCL controller as an external trigger to synchronize with the scanning of the covered spectral region. Spectra are recorded between 1725 and 1570 cm^–1^, using a scan rate of 3 μsteps/int (3 s per scan) by averaging 30 scans per spectrum (total acquisition time = 90 s) and sent to the computer via a data acquisition card (DAQ Card, 6001-USB, National Instruments, Salzburg, Austria) for data processing.

### Automated Measurement Procedure

Prior to the measurement, both channels of the transmission cell are filled as described in the Sample Handling and Injection System section. As a major improvement to our previous work,^16^ where the piezo controller was used only to manually move the interferometer to the quadrature point (QP) at the beginning of each scan, we have now introduced a locking scheme that enables to move automatically the interferometer to the QP and to keep it there throughout the entire spectral scan. This new functionality ensures stability and linearity of the output signal during data acquisition. The locking scheme consists of a PID servo-controlled loop algorithm, programmed and executed in LabVIEW. The PID reads the voltage values at detector D1 and detector D2 and applies the corresponding voltage value to the piezo element such that the mirror moves along the diagonal compensating for optical-path differences and in turn the difference D1 – D2 is kept at zero. The parameters have been set such that drifts can be corrected without interfering in the extraction of the photothermal signals (see also Supporting Information S1). Prior to the measurement, the PID is activated and set to lock the interferometer to the QP. The PID is kept enabled when the EC-QCL is turned on and tuned across its spectral range (1725–1570 cm^–1^) during spectral acquisition and data collection. It is worth noting that this development now permits to perform all data acquisition, including background (*I*_back_) and sample (*I*_sample_) measurement, in an automated way without requiring to manually reset the interferometer back to the same QP between scans. This ensures a constant sensitivity factor during the entire duration of the measurement.

### Data Processing

The collection and storage of the signals as well as the PID feedback is performed by means of an in-house developed LabVIEW GUI. Data are then processed via an in-house developed MATLAB 2020b script. The resulting spectra are a function of the wavelength dependent laser power of the EC-QCL (P) and of the analyte concentration (*c*_m_), as expressed by eq 2. Furthermore, due to absorption from the solvent itself also a power dependent background photothermal signal (*I*_back_) is generated which needs to be subtracted from the recorded photothermal signal of the sample (*I*_sample_). Following eq 3, a power normalized PTS signal from the analyte is calculated for each wavelength, and the corresponding photothermal spectrum can then be qualitatively compared with the corresponding absorbance spectrum of the analyte.
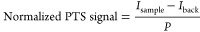
3

A qualitative comparison between the water absorbance spectra recorded with the FTIR (*s*_1_) and photothermal spectra (*s*_2_) was performed to estimate the degree of spectral overlap (*s*_12_) calculated by the following expression:
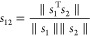
4*s*_12_ can assume a value between 0 (no overlap) and 1 (full overlap).

Spectra were post-processed via additional smoothing using a Savitzky–Golay filter (order: 3, frame length: 21).

### Reference Measurements

Reference measurements for water content have been performed by using a coulometric KF titrator (envirotech CA-21, Düsseldorf, Germany). Each sample has been manually injected and measured 3 times, and the values have been averaged. In general, it takes several minutes to obtain a reference value for a single sample.

Reference absorbance spectra of the water solvent mixtures have been recorded on a Vertex 80v FTIR spectrometer (Bruker Otics, Ettlingen, Germany) furnished with a globar source and a liquid-nitrogen-cooled MCT detector. The spectrometer is constantly flushed with dry air. The samples were manually injected in a transmission cell consisting of two CaF_2_ windows and PTFE spacers of different thickness (480 μm for ethanol and 330 μm both for chloroform and jet fuel). Spectra have been recorded with a resolution of 2 cm^–1^, using an aperture of 1 mm by coadding 100 scans (∼45 s). A Blackman-Harris-3-term apodization function and zero filling factor equal to 2 has been applied, and data have been processed with the OPUS 8.5.29 software from Bruker Optics.

## Results and Discussion

### MZI Working Principle

MZIs sense sub-nanometer phase shifts between their two arms. The two arms of the interferometer are aligned such that a good fringe contrast is achieved, and the interference is maximized. The optical-path difference (Δ*s*) between the two arms of the interferometer can be expressed as:
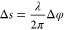
5where λ is the central wavelength of the probe source (632.8 nm) and Δφ is the phase shift. Δφ is directly proportional to the photo-induced refractive index change Δ*n*, occurring as a consequence of sample heating in the analyte channel, as depicted in eq 6:
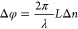
6

Operating the MZI at the QP, where the output intensities onto D1 and D2 are the same, the interferometer exhibits its highest sensitivity and linearity (see Supporting Information S1). At the QP, the phase shift Δφ is an odd multiple of .

### Photothermal Signal Investigation

The EC-QCL operates in CW mode, and a chopper is used to introduce a modulation to the generated thermal wave within the illuminated sample volume leading to a periodical increase and decrease of the sample temperature. In particular, as reported in the literature,^13^ the heating cycle (Light ON) can be expressed as:
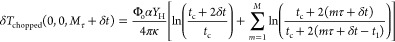
7while the cooling cycle (Light OFF) can be expressed as:
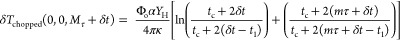
8where Φ_o_ is the total power of the excitation beam, α is the linear absorption coefficient, *Y*_H_is the heat yield, and κ is the thermal conductivity of the medium. *t*_1_ represents the time in which the chopper allows the beam to pass, whereas *t*_2_ represents the time in which the chopper blocks the beam. τ is the sum of *t*_1_ and *t*_2_ and corresponds the inverse of the modulation frequency *f*_mod_. Operating the chopper with a duty cycle of 50%, *t*_1_ and *t*_2_ are equal. *M* is the number of cycles occurred during the experiment, and *t*_c_ is the thermal diffusion time.^13^

**Figure 3:**
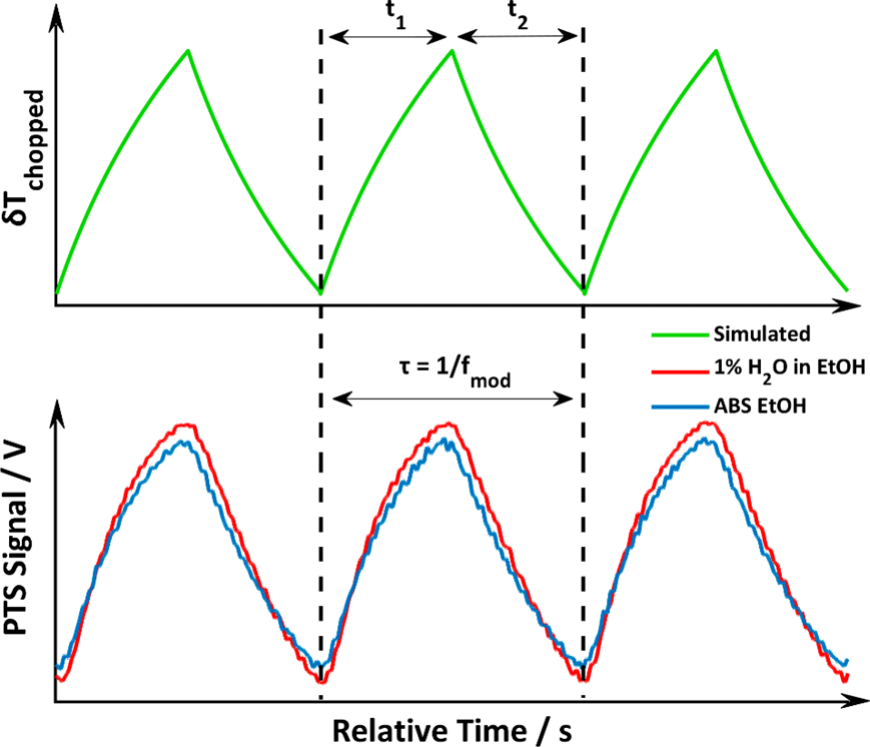
Comparison of simulated (green curve) temperature change and experimental (blue and red curve) PTS signals (excitation@1660 cm^–1^, *f*_mod_=30 Hz, and duty cycle = 50%).

Figure 3 depicts how the photothermal signal is regulated by the modulation frequency *f*_mod_ of the chopped excitation source. As per eqs 7 and 8, the temperature increases for an amount of time equal to *t*_1_ (Light ON) and starts decreasing following a logarithmic trend for a duration of *t*_2_ (Light OFF).

The PTS signals recorded for the background (absolute ethanol) and the sample (1% of H_2_O in absolute ethanol) are depicted in Figure 3 and clearly show a strong contribution of the solvent background absorption at 1660 cm^–1^ to the measured PTS signal. The experimental data exhibit a very good overlap with the simulated results. The EC-QCL is set to emit at 1660 cm^–1^, and the adopted chopper modulation frequency *f*_mod_ is 30 Hz. See Supporting Information S2 for a dedicated study regarding modulation frequency *f*_mod_ influence on the photothermal signal.

### Photothermal Mid-IR Spectra Recorded with the PTS-MZI Spectrometer

A demonstration of the capabilities of the developed spectrometer is given using ethanol, chloroform, and jet fuel as solvents.

### Ethanol

Ethanol, as a protic, polar, and hydrophilic solvent, strongly interacts with water molecules through hydrogen bonds. Hence, water can be dissolved in ethanol at any concentration. A set of dilutions have been prepared and measured with the PTS-MZI spectrometer.

**Figure 4:**
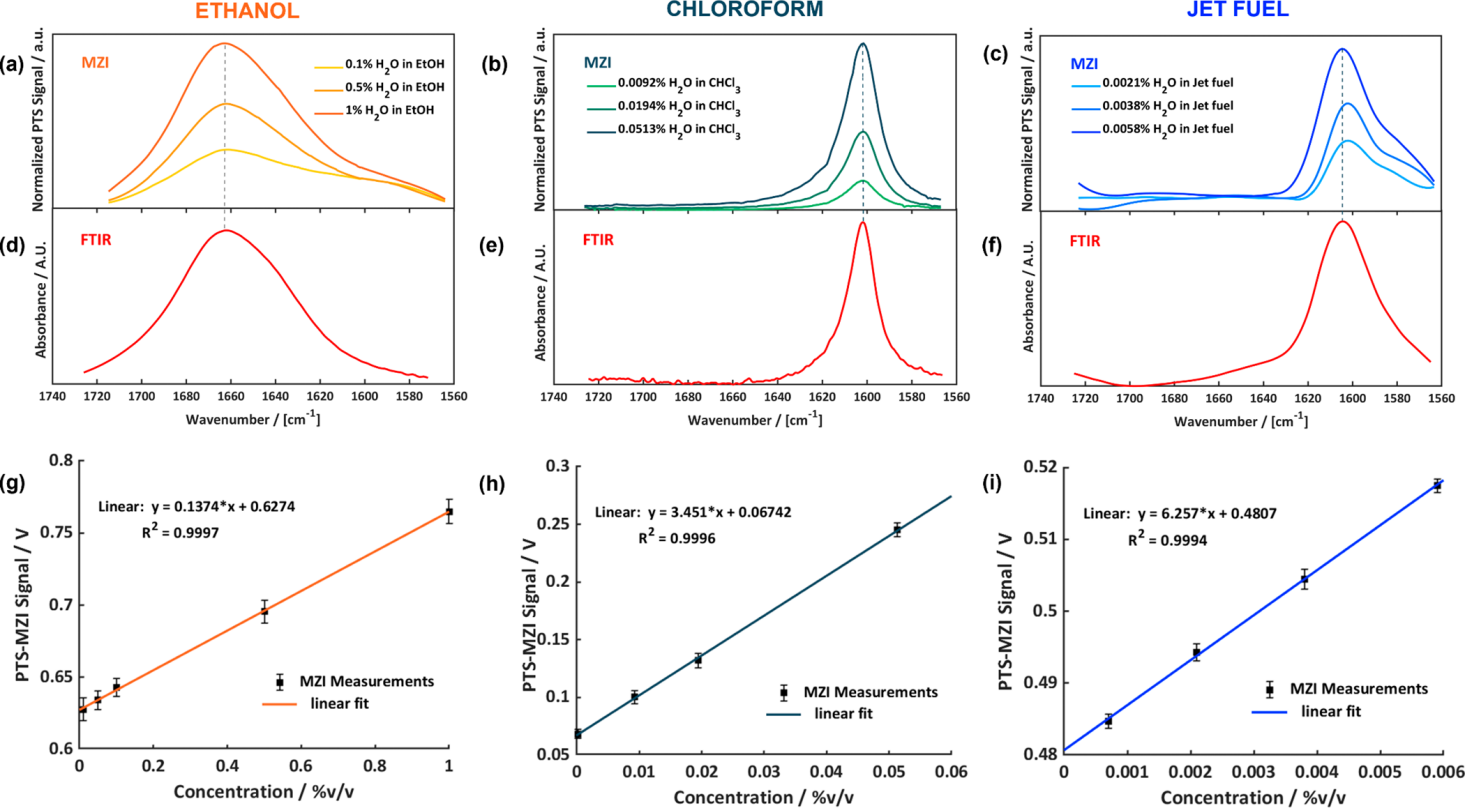
Mid-infrared (mid-IR) spectra of different concentrations of H_2_O in EtOH (a), H_2_O in CHCl_3_ (b), and H_2_O in jet fuel (c) recorded via PT-MZI. Mid-IR spectra of H_2_O in EtOH (d), H_2_O in CHCl_3_ (e), and H_2_O in jet fuel (f) recorded via FTIR. Calibration lines of H_2_O in EtOH (g), H_2_O in CHCl_3_ (h), and H_2_O in jet fuel (i).

The water content already present in the absolute ethanol used for preparing solutions was measured using a coulometric KF titrator and found to be 101.3 ppm. Representative photothermal spectra recorded with the PTS-MZI spectrometer in a concentration range of 0.1–1% H_2_O in ethanol are given in Figure 4a. Moreover, a calibration line, in a concentration range between 0.01 and 1%, is included (Figure 4g).

Water exhibits a strong and broad absorption peak at about 1660 cm^–1^. The slight blue shift when compared to pure water (peak maximum at ∼1645 cm^–1^) of the H–O–H bending mode in the spectra of water–ethanol mixture is due to intermolecular interactions.^25,26^ This effect has recently been reported for water in ethanol by Yu et al.^27^ as well as in other alcohol mixtures.^28,29^ Several studies have been carried out concerning the hydrogen bonding in water–ethanol binary solutions.^30^

The photothermal spectra of water in ethanol are in excellent agreement with the FTIR absorbance spectrum (see Figure 4a,d). The high conformance between the spectra can be seen from the degree of spectral overlap *s*_12_ which was calculated to be 0.99 (see eq 4).

### Chloroform

Chloroform, unlike alcohols, is an aprotic, hydrophobic solvent that is able to retain only a minute amount of water. The solubility of water in CHCl_3_ is only 0.056%.^31^ For that reason, it was used to test the performance of the PTS-MZI spectrometer at very low concentrations.

The bending vibration of water in chloroform has a smaller width (Figure 4b,e), compared to the above-mentioned case of ethanol, and the peak maximum is shifted toward lower frequency (to around 1600 cm^–1^). This is probably due to the absence of hydrogen bond interactions between water and chloroform. Similar results have been reported by Zhou et al.^32^

The FTIR absorption spectrum, depicted in Figure 4e, has been recorded using chloroform dried with molsieve as background and chloroform exposed to water as a sample. The obtained photothermal spectrum is again in excellent agreement with the reference FTIR absorbance spectrum, as corroborated by the correlation coefficient *s*_12_ of 0.99 (see eq 4). The corresponding calibration line covering a concentration range between 0.0001 and 0.05% is shown in Figure 4h.

### Jet Fuel

Jet fuel type A-1 is an aviation fuel suitable for most jet aircrafts, compliant to the international safety requirements. It has been used as a solvent to test the PTS-MZI spectrometer for a typical real-world scenario of the aviation industry in which detecting water represents a daily necessity. Compared to water in ethanol the maximum of the H–O–H bending vibration is shifted toward lower frequencies (∼1608 cm^–1^). The band is slightly broader than the one observed in chloroform (Figure 4c,f). The position of the band maximum thus corresponds to the value of 1608 cm^–1^ found for water in type A-1 jet fuel as reported in the recent study on fuels and biofuels by Łosiewicz.^33^

The FTIR absorption spectrum, reported in Figure 4f, has been recorded using jet fuel dried with molsieve as background and jet fuel exposed to water as a sample. The obtained photothermal spectra matched again the FTIR absorbance spectra as can be judged also by the obtained correlation coefficient *s*_12_ of 0.97. The corresponding linear calibration line covered a concentration range from 0.0007 to 0.0059% (Figure 4i).

### Sensor Calibration, LODs and LOQs

The limit of detection (LOD) and limit of quantification (LOQ) were determined for each solvent under investigation. LOD and LOQ are expressed as:^34^
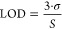
9
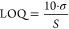
10where σ represents the standard deviation (std) of 30 replicates acquired at or near zero concentration (*I*_back_) and *S* considers the sensitivity that can be derived from the slope of the calibration lines reported respectively in Figure 4g for ethanol, in Figure 4h for chloroform, and in Figure 4i in the case of jet fuel. The linear calibration functions are calculated from the height of the band maxima of the raw PTS-MZI signal, before background removal and optical power normalization. The obtained values are reported in Table 1. See Supporting Information S3 for linearity assessment.

**Table 1:** Results Achieved for Each Solvent, Comparing the Slope of the Calibration Curve (*S*) and Values for Limit of Detection (LOD) and Limit of Quantification (LOQ)

solvent	*S* (V/%)	LOD (%)	LOQ (%)
ethanol	0.14	4 × 10^–2^	0.14
chloroform	3.45	7.4 × 10^–4^	2.5 × 10^–3^
jet fuel	6.26	2 × 10^–3^	6 × 10^–3^

### Influence of the Solvent and Measurements Conditions

The different solvents play a significant role in the achievable LODs and LOQs for detecting water traces in these.

According to eq 2, the magnitude of the PTS-MZI signal depends mainly on three sample dependent quantities, namely: the physical properties (specific heat  and density ρ) of the solvent and the absorption coefficient of the analyte .

Whereas the product ρ · *C*_p_ does not change considerably for the solvents under investigation, several additional factors need to be considered when comparing the obtained analytical figures of merit. The absorption coefficient of water α_a_(ν̃) is strongly influenced by the surrounding environment, which affects both the peak position and the band shape (see Figure 4). As a consequence, the absorption coefficient of water at the wavenumber of maximum band intensity is different which clearly influences sensitivity of analysis.^26,32,35^ The wavelength dependent optical power provided by the EC-QCL also affects the achievable sensitivity across the explored spectral region. Finally, the solvent absorption α_s_(ν̃) itself has a strong influence on the achievable sensitivity.^13^ Solvent absorption and matrix effects limit the available and residual optical power for analyte excitation, reducing in turn the magnitude of the photothermal effect that can be attributed to the analyte. In particular, the solvent absorption coefficients at the maximum of the water absorption band, α_EtOH_(1660 cm^–1^) ∼ 22.33 cm^–1^ and α_CHCl_3__(1600 cm^–1^) ∼ 0.88 cm^–1^ are clearly different. Due to the significantly higher background absorption of ethanol at the measurement wavelength compared to chloroform, less power is remaining for exciting water explaining the strong difference obtained for the respective LODs and LOQs. The solvent also participates in the generation of a thermal gradient hence limiting the sensor linear range for analyte detection. See Supporting Information S4 for a discussion of the encountered differences in the power absorbed by water when detected in ethanol and in chloroform as well as how this affects the slope of the respective calibration lines.

## Conclusions and Outlook

In this study, we have reported for the first time a PID-locked photothermal spectrometer based on an MZI for highly stable and sensitive measurement of solutes on the example of quantifying water in solvents. Using an open–closed system for in situ drying of the organic solvents during measurements, the sample reference (background) spectrum is generated during analysis. By subtraction of the background spectrum from the sample spectrum followed by normalization to the wavelength dependent power of the employed EC-QCL excitation source, photothermal spectra are obtained, which match the corresponding absorbance spectra recorded on an FTIR spectrometer. The developed approach works in polar solvents (ethanol), apolar solvents (chloroform), and also in complex mixtures (jet fuel). Due to the nature of the photothermal signal generation, a dedicated calibration curve for water in each of the studied solvent is required. We have demonstrated that photothermal spectroscopy can achieve highly competitive results in comparison with the well-established KF coulometric technique. However, in contrast to this state-of-the-art technique for quantitation of water, our approach does not require the usage of hazardous substances and can be used for real-time monitoring (<1 min measurement time per spectrum) representing a valid and green alternative to the KF technique (see Supporting Information S5 for GREEnness evaluation according to^36^). The measurements have been carried out using the same conditions for all studied solvents. We expect that the overall system performance can be further refined by optimizing the cell pathlength *L* and the modulation frequency *f*_mod_ for each solvent under investigation. This would improve the noise level for each different matrix and in turn also the resulting LODs and LOQs. Finally, we would like to mention that the detection scheme introduced in this study can most likely be improved by replacing free space optics by fibers and through miniaturization of the whole system. In doing so, noise due to varying water vapor concentration in the optical path can be efficiently minimized as well as the overall noise floor reduced. A final step in this optimization could be a chip-based MZI using integrating waveguides.

## Supporting Information Available

Linear working range, PTS signal dependency on modulation frequency, linearity assessment, solvent contribution, and GREEnness evaluation (PDF)
