# Supplementary material for: Dual-Beam Photothermal Spectroscopy Employing a Mach–Zehnder Interferometer and an External Cavity Quantum Cascade Laser for Detection of Water Traces in Organic Solvents
Source: Anal Chem. 2022 Nov 16;94(47):16353–60. doi: 10.1021/acs.analchem.2c03303 (PMC9716552; doi:10.1021/acs.analchem.2c03303)
Supplement: Supplementary file 1 — ac2c03303_si_001.pdf [file ac2c03303_si_001.pdf]

Supporting Information

Dual-beam photothermal spectroscopy employing a Mach-Zehnder interferometer and an external cavity quantum cascade laser for detection of water traces in organic solvents

Giovanna Ricchiuti,† Alicja Dabrowska,† Davide Pinto, † Georg Ramer† and Bernhard Lendl†, *

† Institute of Chemical Technologies and Analytics, TU Wien, Getreidemarkt 9/164-UPA, 1060, Vienna, Austria

Table of Contents

Linear working range of the PTS-MZI spectrometer……S1

Photothermal signal dependency on the modulation frequency……S2

Linearity Assessment……S3

Photothermal signal – Solvent contribution……S4

GREEnness evaluation……S5

Linear working range of the PTS-MZI spectrometer

To enable accurate quantitation, the analytical signal provided by the PTS-MZI spectrometer needs to show a linear dependence on sample absorption. Hence, even at high laser powers and for concentrated samples, the maximum phase shift needs to stay close to the QP being the part of the interferogram where D1-D2 shows an approximately linear dependence on the phase shift. A demonstration of the linearity of the PTS-MZI spectrometer can be given by estimating the deviation of its output curve, both in terms of the raw signal D1-D2 as well as the extracted demodulated PTS-MZI signal, being the signal demodulated at the applied chopper frequency from a tangential straight line through the QP. In particular, in Figure S1 the D1-D2 and the PTS-MZI Signal for a 1% of H_2O in ethanol, measured with a pathlength of 110 µm, is reported. Even in this "worst-case" scenario of the highest measured concentration (1% H_2O) the signal is in the linear range of the signal response provided by the PTS-MZI spectrometer with a deviation of less than 1% between linear approximation and actual response. This is also still the case when considering baseline drifts occurring within a single spectrum.

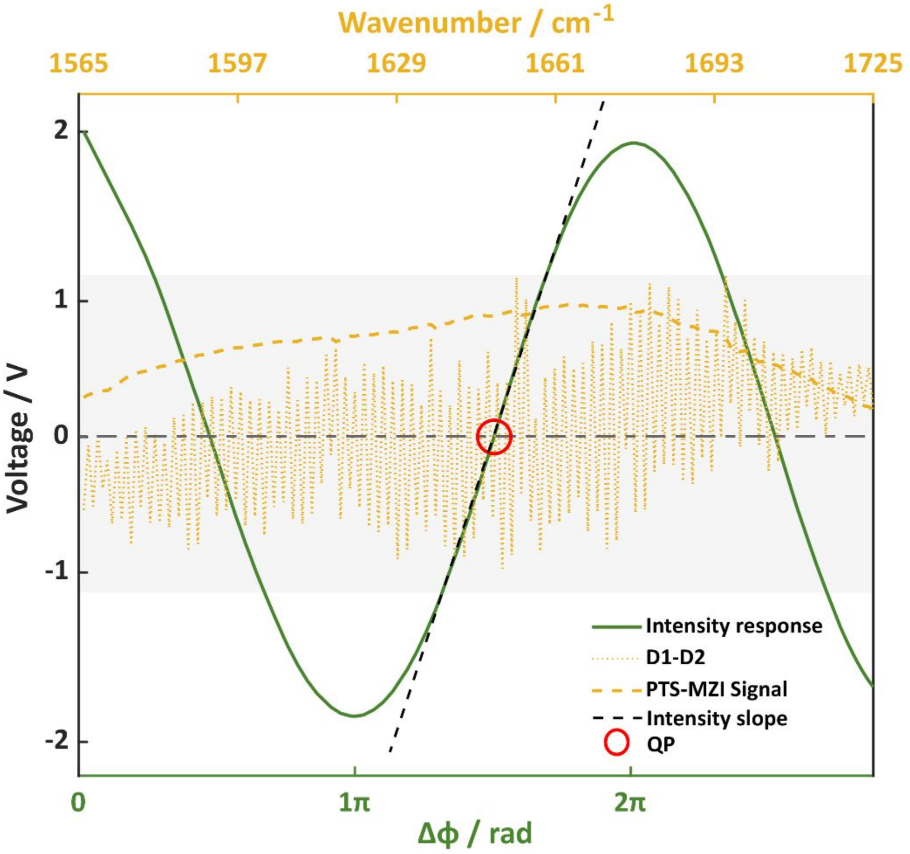

Figure S1. Sensor linearity demonstrated for 1% of H_2O in EtOH. The intensity response (green curve) is the interferometer characteristic of the MZI and the tangent at the zero intercept (quadrature point QP, red circle) is depicted by the black-dashed line. The gray shaded area represents the span of signal intensities where the tangent and the intensity response deviate by less than 1%. The light yellow D1-D2 is the raw signal during the acquisition and the yellow-dashed line is the demodulated PTS-MZI Signal amplitude during the acquisition.

Photothermal signal dependency on the modulation frequency

An investigation on the dependency of the demodulated differential PTS-MZI Signal on the modulation frequency f_{mod} was performed. Both channels of the transmission cell were filled with the solvent (absolute ethanol) and a series of different modulation frequencies were subsequently selected via the optical chopper controller in a range between 30 and 480 Hz. Figure S2 demonstrates that the PTS-MZI Signal scales as an inverse function of f_{mod}.

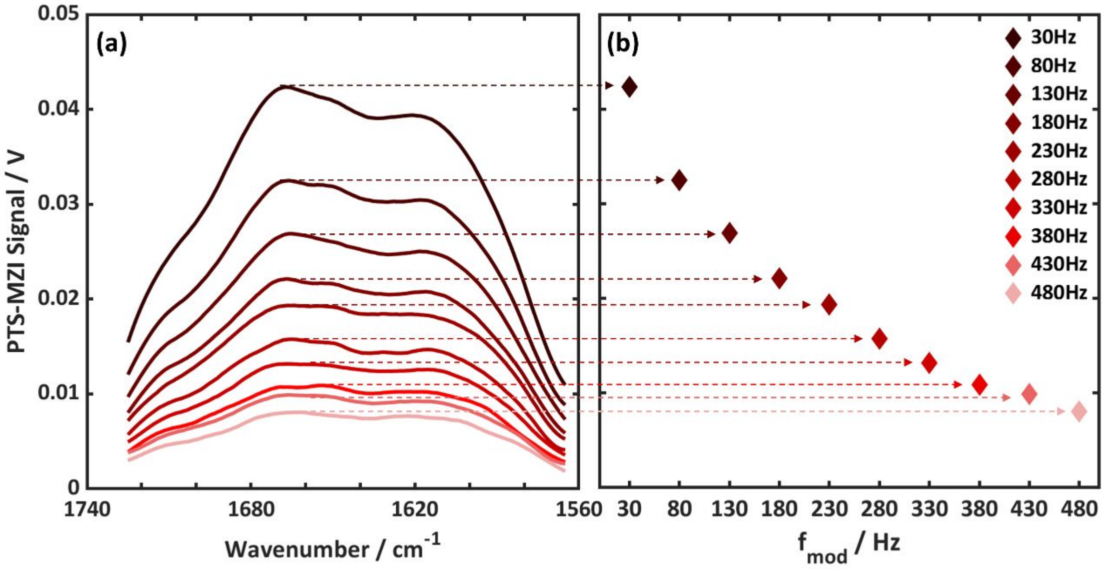

Figure S2. PTS-MZI Signal dependency on the modulation frequency in a frequency range [30-480 Hz] (a). PTS-MZI Signal maxima at each modulation frequency f_{mod} plotted against the different modulation frequency range under investigation shows that the signal scales as $1/f_{mod}$ (b).

Linearity Assessment

Goodness-of-fit test in accordance with Mandel, as defined [1-3]:

$$\hat{F} = \frac{(N-2){\sigma_{y1}}^2 - (N-3){\sigma_{y2}}^2}{{\sigma_{y2}}^2} = \frac{(N-2)\sum_{i=1}^{N} \frac{\left(\overline{y_i} - \widehat{y_{il}}\right)^2}{N-2} - (N-3)\sum_{i=1}^{N} \frac{\left(\overline{y_i} - \widehat{y_{iq}}\right)^2}{N-3}}{\sum_{i=1}^{N} \frac{\left(\overline{y_i} - \widehat{y_{iq}}\right)^2}{N-3}}$$

Where N is the number of the considered calibration point for each solvent (in the case of ethanol 5 calibration points are considered, in both the case of chloroform and jetfuel, 4 calibration points are considered), σ_{y1} denotes the residual standard deviation of the linear calibration function, σ_{y2} denotes the residual standard deviation of the quadratic calibration function, $\overline{y_i}$ represents the experimental measured signal at each x-calibration value, $\widehat{y_{il}}$ denotes the predicted response by the linear model and $\widehat{y_{iq}}$ denotes predicted response by the quadratic model.

Table S1. Mandel goodness-of-fit evaluation. In the first column the solvent under investigation, in the second column the variance of the linear calibration function, in the third column the variance of the quadratic calibration function, in the fourth column the F-table value $\hat{F}_{tab(95\%)}$ at a significance level α = 0,05 and in the fifth column the evaluated goodness-of-fit $\hat{F}$ value

Solvent	${\sigma_{y1}}^2$	${\sigma_{y2}}^2$	$\hat{F}_{tab(95\%)}$	$\hat{F}$

Ethanol	$1.6\cdot10^{-6}$	$2.3\cdot10^{-6}$	$\hat{F}_{tab(95\%)}$ (0.05,1,2)= 18.513	0.05
Chloroform	$3.9\cdot10^{-6}$	$3.5\cdot10^{-6}$	$\hat{F}_{tab(95\%)}$0.05,1,1)= 161.45	1.19
Jetfuel	$1.7\cdot10^{-7}$	$1.7\cdot10^{-7}$	$\hat{F}_{tab(95\%)}$ (0.05,1,1)= 161.45	0.97

$\hat{F}$ is compared with the value from the F-table for the significance level α = 0,05 (see ISO 8466-2:2021 and ISO 8466-2:2001) for f_1 = 1 and f_2=N−3. In the three cases, being $\hat{F} < \hat{F}_{tab(95\%)}$, the linear model is applicable.

Photothermal signal – Solvent contribution

The different sensitivities observed when measuring water in the different solvents merit a further discussion which highlights the peculiarities of indirect photothermal measurements as opposed to absorbance measurements, which are usually evaluated using Beer´s law. In absorption spectroscopy absorbance is calculated as the logarithm of the ratio of two single beam spectra (background and sample). In contrast, in photothermal spectroscopy the intensity normalized background spectrum is subtracted from the intensity normalized sample spectrum to yield the photothermal spectrum of the analyte. Data shown in Figure 4 (See the main document) corroborate that band position and shape of the derived absorbance as well as photothermal spectra are in very good agreement. Furthermore, in the case of absorbance spectroscopy common experience tells that to a very good degree the area of an absorption band is, in a first approximation and excluding analyte solvent interactions, independent of the solvent – even if the solvent has overlapping absorption features. This is because unspecific background absorbance is cancelled when calculating the absorbance spectrum. However, in case of photothermal spectroscopy background absorption has a strong influence on the amplitude of the generated analyte specific photothermal signal. Consequently, different slopes of the respective calibration curves have been obtained. Here we would like to give a rough estimation of the most relevant factors governing the sensitivity of measuring water in different solvents.

In literature the amplitude of the photothermal signal is often expressed as per Eq. (2)[4,5]:

$$\text{PTS} - \text{MZI Signal} \propto \Delta\text{T} = \frac{P(\tilde{v})\ \alpha(\tilde{v})\ L}{\rho C_P V f_{mod}}$$

Eq. (2) applies in regime of small absorption where the absorbed power P_{ABS} can be approximated as $P\alpha L$, assuming $\alpha L \ll 1$.

In the specific case of water in different solvents studied in this work this approximation is not correct as for some solvents $\alpha L > 1$. Furthermore, this equation does not take into account the large thermal mass of the flow cell windows for heat dissipation. However, as a first approximation to estimate the magnitude of the generated photothermal signal we consider the power deposited due to absorption P_{ABS}:

$$P_{ABS} = P(1 - 10^{-\alpha L})$$

The local temperature increase is then mainly dependent on absorbed power P_{ABS}, the density ρ and the heat capacity C_P of the solvent.

In the applications discussed in our paper, two absorbers, the water itself as well as the solvent, contribute to power attenuation when light is passing through the optical pathlength L. The two contributions can be expressed as:

$$P_{WATER,\,ABS}(L) = \frac{\alpha_{WATER}}{\alpha_{WATER} + \alpha_{SOLVENT}} \cdot P_0(1 - 10^{-(\alpha_{WATER} + \alpha_{SOLVENT})L})$$

$$P_{SOLVENT,\,ABS}(L) = \frac{\alpha_{SOLVENT}}{\alpha_{WATER} + \alpha_{SOLVENT}} \cdot P_0(1 - 10^{-(\alpha_{WATER} + \alpha_{SOLVENT})L})$$

As the sensitivity of the spectrometer depends on the slope of the calibration line and assuming that the background contribution remains constant for each sample concentration, we can calculate the ratio between $P_{1,\,ABSCHCl_3}(L)$ and $P_{1,\,ABSEtOH}(L)$, being the power absorbed by the water in the respective solvents considering

an optical pathlength L=110µm, and the absorption coefficients α as determined from absorbance spectra or taken from literature as follows:

$$\frac{P_{WATER,\,ABS_{CHCl_3}}(L)}{P_{WATER,\,ABS_{EtOH}}(L)} = \left(\frac{\frac{\alpha_{H_2O\,+\,CHCl_3}}{\alpha_{H_2O\,+\,CHCl_3} + \alpha_{CHCl_3}} \cdot P_0(1 - 10^{-(\alpha_{H_2O\,-\,CHCl_3} + \alpha_{CHCl_3})L}}{\frac{\alpha_{H_2O\,-\,EtOH}}{\alpha_{H_2O\,+\,EtOH} + \alpha_{EtOH}} \cdot P_0(1 - 10^{-(\alpha_{H_2O\,+\,EtOH} + \alpha_{EtOH})L}} \right) \sim 8.2$$

As chloroform shows negligible absorption within the studied spectral range, when using chloroform as a matrix the full optical power provided by the laser is available to excite the analyte under investigation. That is not the case for ethanol, where most of the available power is attenuated by the solvent itself and in turn less residual power is available to heat up water molecules. Thus, the photothermal effect is expected to be 8 times higher in a chloroform matrix than in an ethanol matrix.

Furthermore, the specific heat capacity of the matrix (i.e. product of its density and heat capacity, ρC_P) describes the amount of absorbed energy required to cause a temperature change. Thus, we get the following ratio to describe the difference in response to heating for ethanol and chloroform

$$\frac{\rho_{CHCl_3}\, C_{P_{CHCl3}}}{\rho_{EtOH}\, C_{P_{EtOH}}} \sim 0.8$$

Finally, we estimate the ratio of temperature change ΔT for the same amount of analyte in the photothermal experiment for chloroform and ethanol:

$$\frac{\Delta T_{CHCl_3}}{\Delta T_{EtOH}} = \frac{P_{WATER,\,ABS_{CHCl_3}}(L)}{P_{WATER,\,ABS_{EtOH}}(L)} / \frac{\rho_{CHCl_3}\, C_{P_{CHCl3}}}{\rho_{EtOH}\, C_{P_{EtOH}}} \sim 10.25$$

Thus, this simple consideration provides a good explanation why the slope of the calibration curve for water depends on the matrix. A more detailed theoretical analysis of all influence factors contributing to the observed photothermal signal – while possible - is beyond the scope of this work.

GREEnness evaluation

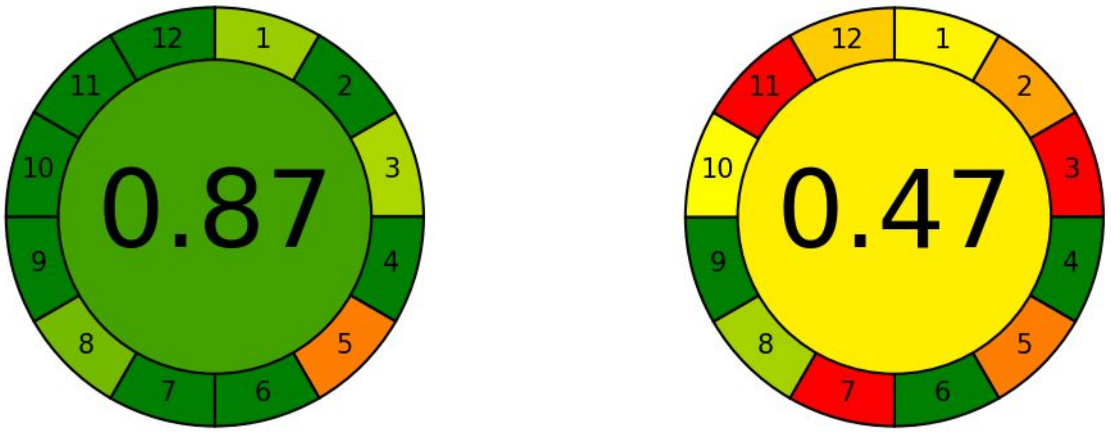

Figure S3. Results of evaluation according to the Analytical GREEnness metric approach[6] for (A) on-line PTS-MZI spectrometer and (B) off-line KF analysis. The clock-like graphs include 12 segments, representing the SIGNIFICANCE criteria. The performance of each principle is represented by a green-yellow-red color range, whereas the weight-importance is reflected by the width of each segment. The overall performance of the methods is expressed by the color and score (0=worst, 1=best) in the middle.

Table S2. Selected options for the Analytical GREEnness evaluation

Parameter	PTS-MZI spectrometer		Karl Fischer	
	Input	Score	Input	Score
1. Sampling procedure	On-line	0.7	Off-line	0.48
2. Amount of sample in g or mL	0.04 (independent on the concentration to detect)	1.0	10 (for the lowest H_2O concentration)	0.32
3. Position of analytical device	On-line	1.0	Off-line	0.0
4. Sample preparation steps	3 or fewer	1.0	3 or fewer	1.0
5. Automation, miniaturization	Semi-automatic, not miniaturized	0.25	Semi-automatic, not miniaturized	0.25
6. Derivatization	none	1.0	none	1.0
7. Amount of waste in g or mL	0	1.0	200	0.0
8. Number of analytes per run, sample throughput per hour	1, 30	0.68	1, 20	0.61
9. Most-energy intensive	FTIR	1.0	Titration	1.0
10. Type of reagents	No reagents	1.0	Some reagents are bio-based	0.5
11. Toxic reagents or solvents	0	1.0	Yes, 200	0.8
12. Threats	None	1.0	Toxic, Flammable, Corrosive	0.4

References

(1) ISO 8466-1:2021 Water Quality — Calibration and Evaluation of Analytical Methods — Part 1: Linear Calibration Function; Vol. ISO 8466-1:2021(E).

(2) Andrade, J. M.; Gómez-Carracedo, M. P. Notes on the Use of Mandel's Test to Check for Nonlinearity in Laboratory Calibrations. Anal. Methods **2013**, 5 (5), 1145. https://doi.org/10.1039/c2ay26400e.

(3) L. A. Currie, K. D. Guidelines for Calibration in Analytical Chemistry. Part 1. Fundamentals and Single Component Calibration. Pure Appl. Chem. **1998**, 70 (4), 993–1014. https://doi.org/10.1351/pac199870040993.

(4) Mazzoni, D. L.; Davis, C. C. Trace Detection of Hydrazines by Optical Homodyne Interferometry. Appl. Opt. **1991**, 30 (7), 756. https://doi.org/10.1364/AO.30.000756.

(5) Bialkowski, S. E. Photothermal Spectroscopy Methods for Chemical Analysis; John Wiley & Sons, 1996.

(6) Pena-Pereira, F.; Wojnowski, W.; Tobiszewski, M. AGREE—Analytical GREEnness Metric Approach and Software. Anal. Chem. **2020**, 92 (14), 10076–10082. https://doi.org/10.1021/acs.analchem.0c01887.
